# Sponge-Inspired Dibromohemibastadin Prevents and Disrupts Bacterial Biofilms without Toxicity

**DOI:** 10.3390/md15070222

**Published:** 2017-07-12

**Authors:** Tiffany Le Norcy, Hendrik Niemann, Peter Proksch, Karen Tait, Isabelle Linossier, Karine Réhel, Claire Hellio, Fabienne Faÿ

**Affiliations:** 1 Laboratoire de Biotechnologie et Chimie Marines, Institut Universitaire Européen de la Mer, Université de Bretagne-Sud, 56100 Lorient, France; tiffany.le-norcy@univ-ubs.fr (T.L.N.); isabelle.linossier@univ-ubs.fr (I.L.); karine.rehel@univ-ubs.fr (K.R.); fabienne.fay@univ-ubs.fr (F.F.); 2 Institute of Pharmaceutical Biology and Biotechnology, Heinrich-Heine-University Düsseldorf, 40225 Düsseldorf, Germany; hendrik-niemann@web.de (H.N.); peter.proksch@hhu.de (P.P.); 3 Plymouth Marine Laboratory, Plymouth PL1 3DH, UK; KTAIT@pml.ac.uk; 4 Biodimar, LEMAR UMR 6539, Institut Européen de la Mer, Université de Bretagne Occidentale, 29200 Brest, France

**Keywords:** biofouling, biofilm, bastadin derivative, microfouling, quorum sensing, sponge

## Abstract

Since the banning of several families of compounds in antifouling (AF) coatings, the search for environmentally friendly AF compounds has intensified. Natural sources of AF compounds have been identified in marine organisms and can be used to create analogues in laboratory. In a previous study, we identified that dibromohemibastadin-1 (DBHB) is a promising AF molecule, leading to the inhibition of the activity of phenoloxidase, an enzyme involved in the attachment of mussels to surfaces. This paper describes the activity of the DBHB on biofilm formation and its detachment and on bacterial adhesion and communication: quorum sensing. DBHB has an anti-biofilm activity without affecting adhesion of marine and terrestrial bacteria at a dose of 10 µM. Moreover, DBHB activity on quorum sensing (QS) is demonstrated at doses of 8 and 16 µM. The activity of DBHB on QS is compared to kojic acid, a quorum sensing inhibitor already described. This compound is a promising environmentally friendly molecule potentially useful for the inhibition of microfouling.

## 1. Introduction

All surfaces immersed in aquatic environments are rapidly colonized by organisms (microfoulers and macrofoulers); this natural phenomenon is defined as biofouling [1].

It is occurring in three main sequential stages: immediately after immersion, surfaces are covered by dissolved organic molecules which then stimulate subsequent adhesion of microorganisms such as bacteria, fungi and microalgae; these organisms constitute the microfouling. It acts as attractant for the adhesion of macrofoulers such as macroalgae (zygote and/or spores) and invertebrate larvae [2,3,4].

Within biofilms, bacteria are usually present in higher densities than in other microorganisms. These bacterial communities are constituted by sessile bacteria, which are adhered to each other and/or to the surface, with the adhesion being facilitated by the production of exopolysaccharides (EPS) [5]. The formation of microbial biofilm is a complex process which involves communication such as quorum sensing (QS). QS is a microbial signaling system which allows for the regulation of population behaviors such as the production of virulence factors [6] or the formation of biofilm [7]. The QS system involves small molecules named autoinducers (AI). These molecules, linked to the protein receptor, induce the expression of several genes involved in population behaviors such as biofilm formation, motility or pigment production. One of the family of QS signal molecules are acyl-homoserine lactone (AHL) type molecules. This bacterial communication was first described in a marine bacteria *Vibrio fisheri* [8]. Later, similar signaling systems were discovered in other bacterial species. Two systems are described: in Gram negative bacteria, AHL-like communication molecules are present, whereas in Gram positive bacteria, other molecules (autoinducer-2: pheromones) are identified [9]. It was described that QS plays an important role in numerous processes governing biofilm formation and organization. Waters et al., demonstrated that when *Vibrio cholera* was in low cell density, the phosphorylation of LuxO inhibited an mRNA which induced the expression of a protein inhibiting the C-di-GMP. The C-di-GMP, an intracellular messenger, has already been shown to be involved in bacterial biofilm formation [7]. In the same way, *P. aeruginosa* mutated on the *las*I gene induced a biofilm 20% finer than the wild type [10]. The discovery of a molecule which could interfere with the QS and especially with the biofilm formation would be a promising candidate for new antifouling (AF) compound [11] in replacement of current AF compounds used in paint, such as heavy metals or biocides.

Some compounds have attracted wide-spread attention for their ability to interact with QS. For example, molecules of the furanone family are able to interfere with bacterial communication [12,13]. These compounds possess a chemical structure similar to the structure of AHL [14]. Other molecules, some enzymes (lactonase or acylase) could be used to inhibit QS [15]. For example, an enzyme named Aiia is able to inhibit the QS by the degradation of the lactone ring of AHL, which is involved in the recognition of the receptor [16].

The environment is a source of inspiration for many innovations. In nature, numerous marine organisms which are fixed and/or moving slowly are able to inhibit biofouling [17,18]. Numerous bioactive substances have been isolated from marine organisms such as algae and sponges [19,20]. Such compounds contribute, in most cases, to the chemical defense against predation, or to the inhibition of adhesion and growth of pathogens and epibionts. Sponges are pre-eminent producers of bioactive secondary metabolites with antibacterial, antifungal or anti-inflammatory activities [21,22,23]. From natural molecules, it is also possible to synthesize analogues, which possess equivalent or even stronger activities, due to the addition of specific chemical groups [24,25,26].

Within this study, we decided to focus on the potential of bastadins as AF inhibitors. Bastadins have been previously isolated from a marine sponge *Ianthella basta*. Thirty natural bastadins were characterized and screened for their AF potencies: it was highlighted that some of these compounds were able to inhibit barnacle adhesion and adhesives synthesis by mussels [27,28].

**Figure 1:**
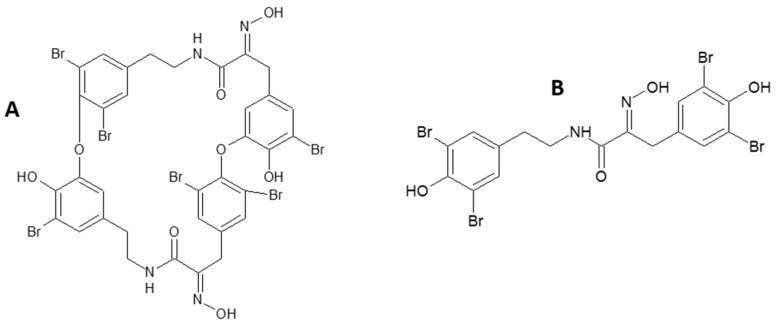
Chemical structure of a natural bastadin (**A**) and the hemibastadin analogue: Dibromohemibastadin-1 DBHB (**B**).

Bastadins are composed of a brominated tyrosine and a brominated tyramine unit linked by a peptide bond in order to form a hemibastadin unit (Figure 1A). The tyrosine moiety furthermore bears an oxime function. Two hemibastadins linked by covalent bonds give rise to a bastadin. Within this study, we focused on the characterization of the activity of a hemibastadin analogue named dibromohemibastadin-1 (DBHB) (Figure 1B). DBHB consists of a hemibastadin unit composed by brominated tyrosine and a likewise brominated tyramine unit; its synthesis was previously described by Bayer et al. in 2011 [27]. DBHB is selected and evaluated in our study because this compound has already been described as an inhibitor of macroorganisms (barnacles and mussels) adhesion [28,29] without toxicity. However, its activity on microorganisms has never been described. Microorganisms are important in biocorrosion, and these organisms can develop resistance against biocides, so it is important to identify new molecules and strategies to limit biofouling. Ortlepp et al. in 2007 published data about DBHB activity toward cyprid larvae of *Barnacle improvisus* and nauplii of *Artemia salina*: DBHB induced an inhibition of the larval settlement at 10 µM without mortality and did not cause mortality of the brine shrimp nauplii when used at 10 µM [29]. The importance of the oxime function and bromine atoms for AF activity was also highlighted. Bayer et al. in 2011, showed that DBHB inhibits phenoloxydase (enzyme involved in adhesive production by *Mytilus* sp.) activity with an IC_50_ of 0.84 µM [27].

The aim of our work is to further characterize the activity of DBHB, with a focus on marine and terrestrial bacterial adhesion and biofilm formation. These experiments have been realized in dynamic conditions, in a flowcell system, established by Tolker-Nielsen [30]. The molecule has also been tested on bacterial communication to determine its mode of action.

## 2. Results

### 2.1. Anti-Bacterial Activity

To determine the antibacterial property of DBHB, the activity of the molecule was evaluated on the growth of four bacteria, three marine bacteria (*Paracoccus* sp. 4M6, *Pseudoalteromonas* sp. 5M6 and *Vibrio* sp. D66) isolated in the Gulf of Morbihan (south Brittany) [31] and a terrestrial bacterium *Pseudomonas aeruginosa* PAO1 used as reference. DCOIT was used as positive control, it is the active compound of the seanine^®^ [32].

**Figure 2:**
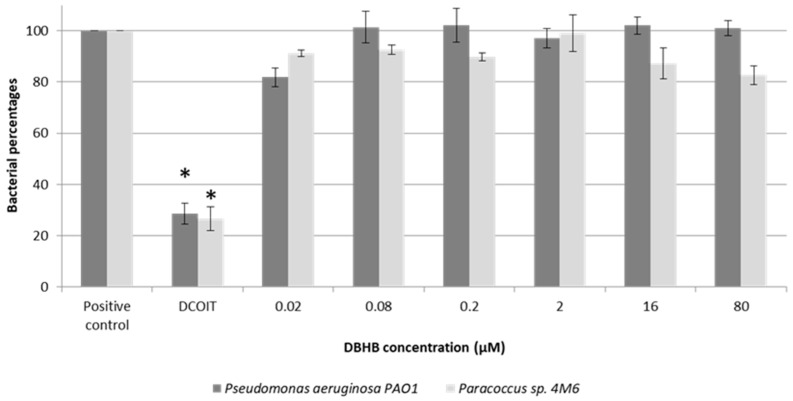
Screening of dibromohemibastadin-1 (DBHB) at six concentrations on two bacteria: *Paracoccus* sp. 4M6 and *Pseudomonas aeruginosa* PAO1 (* *p* < 0.01); the bar represents the standard deviation.

Results do not show any statistical differences (ANOVA, *p* > 0.05) unlike to the addition of the DCOIT, which induced an inhibition of the bacterial percentage by a factor of 4 (Figure 2). The inhibition of bacterial growth by the DCOIT is statistically different to the control (ANOVA, *p* < 0.01).

The anti-bacterial test showed that DBHB does not affect the bacterial growth at doses between 0.02 and 80 µM. DBHB is not toxic for the bacteria studied.

To evaluate the activity of the compound DBHB on bacterial adhesion and biofilm, the concentration tested varied from 2 to 16 µM. This range of concentration was selected for the next experiments because these concentrations were high enough for activity evaluation.

### 2.2. Impact of DBHB on AHL Production

Amongst the four strains studied (PAO1, 4M6, 4J6 and 5M6), three were shown to be able to produce AHLs. However, no AHL was identified in the supernatant of *Pseudoalteromonas* sp. 5M6. The AHL identification is presented in Table 1.

The two bacteria gram negative *P. aeruginosa* PAO1 and *Paracoccus* sp. 4M6 produce three or four different AHLs. For the other gram negative bacterium, *Pseudoaltermonas* sp. 5M6, no AHL was identified. It is probable that this bacterium produces another kind of communication molecule. For the last bacterium, *Bacillus* sp. 4J6, in gram positive bacterium, other molecules of communication have been identified in the literature (Autoinducer-2, AI-2) [33,34].

The addition of DBHB does not modify the AHL production, chromatograms of *P. aeruginosa* PAO1, *Paracoccus* sp. 4M6; and *Pseudoalteromonas* sp. 5M6, with or without DBHB, were similar.

### 2.3. Anti-Adhesion Activity

**Figure 3:**
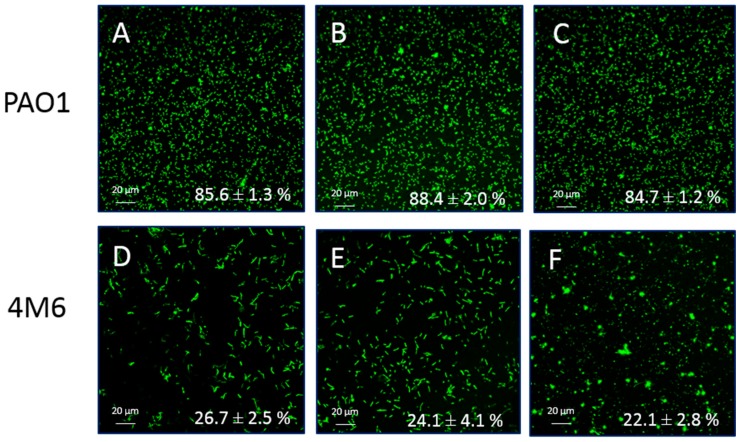
Confocal laser scanning microscopy observations of the bacterial adhesion with syto9^®^ after 2 h with addition of DBHB at 16 µM in two conditions (**A**: PAO1 control, **D**: 4M6 control, **B**,**E**: addition of DBHB to the bacterial suspension for PAO1 and 4M6, **C**,**F**: conditioning of the adhesion surface for PAO1 and 4M6); the overlap percentage (%) is added on observations, the ± represents the standard deviation.

DBHB activity was evaluated on the adhesion of the marine bacterium *Paracoccus* sp. 4M6 and of the terrestrial bacterium *Pseudomonas aeruginosa* PAO1. The molecule activity was determined in the flowcell system [30]. The addition of DBHB in both conditions (within the bacterial growth media or by conditioning the adhesion surface) did not induce any inhibition or activation of the adhesion of *P. aeruginosa* PAO1 or *Paracoccus* sp. 4M6 in comparison to the control. Figure 3 summarizes the observations obtained by Confocal laser scanning microscopy (CLSM) on the adhesion of *P. aeruginosa* PAO1 and *Paracoccus* sp. 4M6 on the glass slide.

It was also observed that the strain *P. aeruginosa* PAO1 adhered far more than the bacterium *Paracoccus* sp. 4M6, there was a factor of 4 between the overlap percentages of *P. aeruginosa* PAO1 and *Paracoccus* sp. 4M6. This difference of overlap percentage was statistically different (*p* < 0.01) and could be explained by the different ability of the bacteria to adhere.

### 2.4. Anti-Biofilm Activity

#### 2.4.1. Biofilm Formation

The impact of DBHB was observed on the biofilm formation. The activity of the molecule was tested on Gram negative bacteria, which produce AHL (*Paracoccus* sp. 4M6 and *P. aeruginosa* PAO1), on a gram negative bacteria and which did not produce AHL (*Pseudoalteromonas* sp. 5M6), as well as on a Gram positive bacterium (*Bacillus* sp. 4J6). AHL production was determined after an extraction and a LC–MS–MS dosage (Data are not shown) [35].

**Figure 4:**
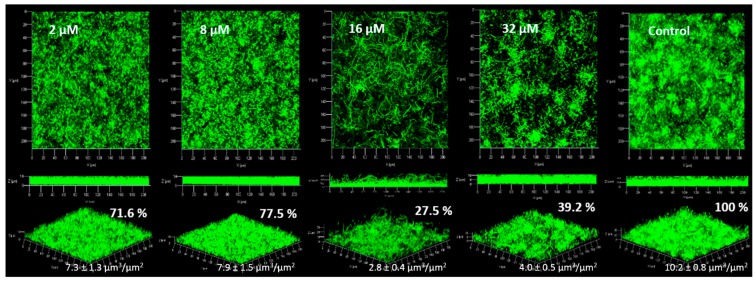
Observation of *P. aeruginosa* PAO1 biofilms (syto9^®^) with addition of DBHB at four concentrations (2, 8, 16 and 32 µM) in the medium growth flow, biovolumes (µm^3^/µm^2^) and biofilms percentages (%) are presented; the ± represents the standard deviation.

For the strain *P. aeruginosa* PAO1, observations obtained in CLSM are shown in Figure 4.

**Figure 5:**
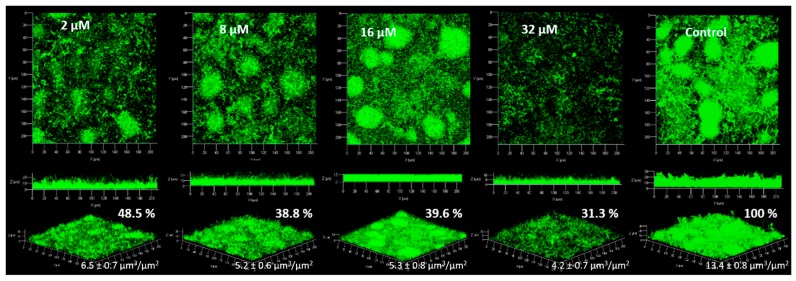
Observation of *Paracoccus* sp. 4M6 biofilms (syto9^®^) with addition of DBHB at four concentrations (2, 8, 16 and 32 µM) in the medium growth flow, biovolumes (µm^3^/µm^2^) and biofilms percentages (%) are presented; the ± represents the standard deviation.

For *P. aeruginosa* PAO1, the inhibition of the biofilm was statistically different from the control only at 16 and 32 µM. At 2 and 8 µM the biofilm was inhibited by only a factor of 1.3. The standard deviations at 2 and 8 µM DBHB was higher than those observed for 16 and 32 µM DBHB, this explains why the biofilm inhibition was not statistically different after analysis with the ANOVA and Tukey tests. During these experiments, the strain *P. aeruginosa* PAO1 at times formed filaments in biofilm and these filaments could modify the biofilm structure and the biovolume associated. The modification of the biofilm structure in *P. aeruginosa* PAO1 could be linked to environmental factors (temperature, pH…). This is why the activity of biofilm inhibition is not the same for the different concentrations evaluated; in flowcell system, it was not possible to control the filament formation. The activity of the compound DBHB was evaluated towards a second strain, a Gram negative bacteria producing AHL: the marine bacterium *Paracoccus* sp. 4M6 (Figure 5).

For the strain *Paracoccus* sp. 4M6, the addition of DBHB to the growth medium induced an inhibition of the biofilm which was significantly different from the control at the four tested concentrations. The biovolumes of *Paracoccus* sp. 4M6 biofilm were 2.1, 2.6, 2.5 and 3.2 times lower than the control for the addition of DBHB at 2, 8, 16 and 32 µM respectively. The similarity of these two bacteria is their ability to produce AHL and their membrane composition, as both are Gram negative bacteria. DBHB is an anti-biofilm compound, but its activity is not permanent. If the molecule is diluted or removed, the biofilm is formed and the biovolume is not significantly different from the control (*p* > 0.05). To inhibit biofilm formation, DBHB has to be in contact with the bacteria.

In order to determine the spectrum of activity of DBHB, a marine bacterium which did not produce AHL (*Pseudoalteromonas* sp. 5M6) and a marine Gram positive bacterium (*Bacillus* sp. 4J6) were also tested and compared (Table 2).

The addition of DBHB in the growth medium flow at 16 and 32 µM did not significantly disturb the biofilm formation of *Bacillus* sp. 4J6 and *Pseudoalteromonas* sp. 5M6. Biovolumes of *Bacillus* sp. 4J6 and *Pseudoalteromonas* sp. 5M6 biofilms were not different from the control condition (about 20 µm^3^/µm^2^ for both bacteria with or without addition of DBHB). In this study, the molecule DBHB seemed to have a specific action on Gram negative bacteria which produce AHL, but more strains should be tested to confirm this result. As it was described that Gram negative bacteria are dominant in the marine environment, it is interesting to have an antibiofilm property on dominant bacteria in seawater [36].

#### 2.4.2. Biofilm Degradation

The second way to evaluate the ability of DBHB to interfere with the biofilm’s behavior was to observe the impact of the compound on a biofilm already formed. DBHB was tested on two strains: *Paracoccus* sp. 4M6 and *P. aeruginosa* PAO1 (Table 3).

After a contact time of two hours between the bacterial biofilm and DBHB, the biofilm was observed. Biofilms that had already been formed were degraded by the addition of the DBHB in the channel flowcell. There was a factor of inhibition of 3 between the biovolume of the control biofilm (with biovolumes values of 12.9 ± 1.9 µm^3^/µm^2^ for 4M6 and 14.6 ± 1.3 µm^3^/µm^2^ for PAO1) and the biovolume after the contact with the DBHB (4.6 ± 1.1 µm^3^/µm^2^ for 4M6 and of 4.5 ± 0.4 µm^3^/µm^2^ for PAO1). These results were proved to be significantly different by a ANOVA (*p* < 0.01) test in comparison with the control.

To validate the activity of DBHB on biofilm formation without an interaction on the bacterial viability, a staining with syto9^®^ and sytoxRed^®^ was performed.

The CLSM observations and the biovolume measurements showed that the addition of DBHB did not induce cells mortality within the biofilm. The percentage of altered cells tagged in red concomitantly with biovolume value (Table 1) indicated that DBHB did not have toxicity against those bacteria. Indeed, the biovolume of dead cells between the control and the biofilm after addition of DBHB were not significantly different (ANOVA and Tukey tests).

### 2.5. Anti-Quorum Sensing Activity

Continuing the demonstration of DBHB activity on biofilm formation, another test using a biosensor, *E. coli* pSB401, was performed in order to determine further biological properties of the compound on bacterial communication. In presence of AHL, the plasmid pSB401 containing QS genes in *E. coli* induces a luminescence which was possible to quantify. If DBHB has an impact on QS genes (*lux*CDABE), the luminescence production would be inhibited by the biosensor *E. coli* pSB401.

**Figure 6:**
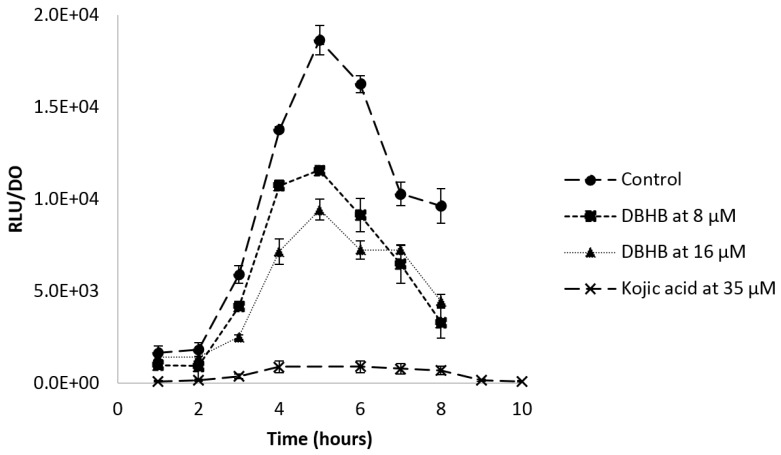
Ratio of the luminescence production and the optical density at 600 nm of the biosensor *E. coli* pSB401 with and without DBHB at 8 and 16 µM; rods represent the standard deviation.

The luminescence production controlled by the recognition between AHL and the receptor is presented Figure 6. An inhibition of the luminescence was observed when the biosensor was in contact with DBHB. The inhibition appeared to be proportional to the concentration of DBHB and the molecule did not alter *E. coli* growth. The control displayed a classical curve of recognition between AHL and the receptor inducing the luminescence. The addition of DBHB at 8 and 16 µM inhibited the luminescence and the maximum values were respectively 1.7 and 2 times lower than those of the control. This inhibition is statistically different from the control (ANOVA and Tukey, *p* < 0.01). When using kojic acid, which is already known as a quorum sensing inhibitor, the inhibition was 20 times lower, as compared to the control.

**Figure 7:**
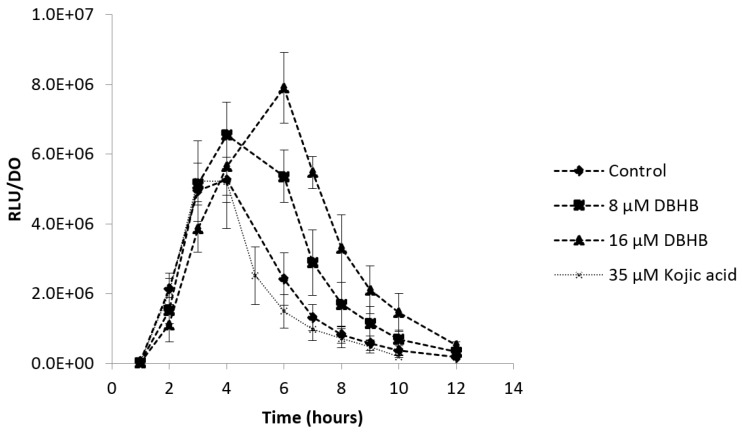
Ratio of the bioluminescence production and the optical density at 600 nm of the biosensor *V. harveyi* JAF548 with and without addition of DBHB at 8 and 16 µM; rods represent the standard deviation.

To validate the anti-quorum sensing property of DBHB and the absence of interaction of the compound with the luminescence production, another biosensor, *V. harveyi* JAF548, was used. For this second biosensor, the bioluminescence production is not dependent of AHL presence. If the luminescence produced by *V. harveyi* JAF548 is inhibited, the molecule would not interact on QS genes but on the luminescence biosynthesis. Figure 7 presents the ratio between the bioluminescence emission and the optical density of *V. harveyi* JAF548 for the control condition and after the addition of DBHB at doses of 8 and 16 µM.

The biosensor *V. harveyi* JAF548 validates the absence of activity of DBHB on the bioluminescence production, confirming the anti-quorum sensing property of DBHB. At the maximum bioluminescence (4 h of growth), the production was not significantly different between the control condition and the addition of DBHB (ANOVA, *p* > 0.05).

## 3. Discussion

### 3.1. Anti-Bacterial Activity

DBHB is not toxic toward the bacteria studied. The absence toxicity is an important factor in order to allow the utilization of a new compound in an antifouling coating to be used in the natural environment. Nowadays, in order to authorize a new biocide, the molecule must have numerous requirements in order to obtain a marketing authorization, such as an absence of toxicity against referent organisms, and not be bioaccumulable. In Europe, a regulation on biocidal products (RBP) is established for the validation and use of biocides [37].

### 3.2. Impact of DBHB on AHL Production

Data show that DBHB does not impact the AHL production by *P. aeruginosa* PAO1 and *Paracoccus* sp. 4M6. Some quorum sensing inhibitor can degrade or perturb the AHL synthesis. For example, some bacteria produce enzymes such as lactonase or acylase, which can degrade AHL [15]. Without AHL, the bacterial communication is inhibited. By targeting the QS, it is possible to inhibit bacterial adhesion or biofilm maturation, which are important steps in microfouling [38].

### 3.3. Anti-Adhesion Activity

Experiments realized to assess the potential interaction of the DBHB on the bacterial adhesion, showed that no significant difference from the control was obtained for the bacteria *Paracoccus* sp. 4M6 and for *Pseudomonas aeruginosa* PAO1. DBHB cannot inhibit the bacterial adhesion.

Other experiments not presented is this study showed that DBHB did not modify bacterial motility of *P. aeruginosa* PAO1: swimming, swarming and twitching. For this motility processes, two kinds of organelles, pili and flagellum, were used. Studies published that these organelles are involved in bacterial adhesion and biofilm structuration [39,40]. These results confirmed that DBHB did not impact cellular mechanism allowing the bacterial adhesion.

### 3.4. Anti-Biofilm Activity

For both strains *Paracoccus* sp. 4M6 and *P. aeruginosa* PAO1, DBHB led to an interesting activity on biofilm formation and biofilm desintegration. The marine strain *Paracoccus* sp. 4M6 was more sensitive than the terrestrial bacterium *P. aeruginosa* PAO1. From tested conditions in this study, DBHB has a specific activity towards Gram negative bacteria which produce AHL.

Gram negative bacteria possesses a complex cell wall, the DBHB could target a specific compound of the Gram negative cell wall which is not present in Gram positive bacteria, such as a lipopolysaccharide; it could cross the bacteria wall by porin protein [41]. In Gram negative bacteria, the outer membrane protects bacteria against antibiotics, detergents or other molecules [42]. It is an interesting result for antifouling application because, in the marine environment, Gram negative bacteria are dominant [36].

DBHB seems also able to target bacterial strains which produce bacterial signal molecule such as AHL; it is possible that this molecule can interfere in quorum sensing.

DBHB was derived from natural bastadins isolated from the marine sponge *Ianthella basta*. It was not surprising to find antibiofilm compounds in marine sponge; these organisms need to produce chemical defenses for their survival [43], against predation or for space and competition for resources [44,45]. Two classes of marine sponge compounds were previously identified as antibiofilm molecules without interaction on the bacterial viability, which include terpenoids [46] and the pyrrole imidazoles [47]. Some of these compounds are presented in Table 4.

Natural compounds which have this type of activity are a source of numerous analogues with biological activities. In this study, the bastadin analogue DBHB was found to be a promising antifouling compound, because this molecule can inhibit biofilm formation without interacting with bacterial growth. DBHB has a simple structure and can be easily synthesized.

DBHB is not the first compound exhibiting bromine atoms that show antibiofilm properties. Other studies have confirmed the antibiofilm property of brominated compounds, for example, in 2008 Huigens et al. showed that the increase of brominated groups in a given molecular structure induced a stronger antibiofilm activity [54]. In another study of Andjouh et al. in 2016, the authors demonstrated the importance of bromine groups for the antifouling performance [55].

An example to consider is the compound oroidin, isolated from the sponge *Agelas oroides*. The molecule has been described as an inhibitor of bacterial adhesion [56]. Forte et al. in 2009, showed that oroidin can inhibit *P. aeruginosa* biofilm with IC_50_ of 190 µM. Structurally, oroidin and DBHB are similar, with two aromatic cycles and bromines. By comparing, IC_50_ of these two molecules on the inhibition of *P. aeruginosa* biofilms, DBHB appeared to be twenty times more active than oroidin [57].

Another explanation of DBHB effect could be an interaction with an intracellular pathway, such as with the C-di-GMP. This intracellular messenger is involved in biofilm formation and dispersion. In *P. aeruginosa*, the level of C-di-GMP induced the extracellular matrix synthesis by the activation of *pel* and *psl* genes [58].

### 3.5. Anti-Quorum Sensing Activity

The study with the biosensor *E. coli* pSB401 demonstrated an anti-QS property of DBHB. The addition of DBHB induces a reduction of the luminescence production; however, this inhibition is not similar to the activity of kojic acid. Kojic acid is described as an inhibitor of QS, which is explained by chemical similarities between AHL and kojic acid. The latter compound was identified as an AHL analogue. Kojic acid can bind to QS receptors such as RhlR, LasR or LuxR and block the communication system [59]. The difference on the inhibition of the bacterial communication by DBHB or acid kojic could be explained by the mode of action of the molecule. It is known that kojic acid possesses a direct activity on QS, as the molecule is an antagonist of AHL, while DBHB could interact on a step of regulation in the QS, which does not induce a total inhibition of the luminescence produced by the biosensor. With these results, we can hypothesize that DBHB does not interact in the same way as kojic acid does on the AHL receptor.

The second biosensor, *V. harveyi* JAF548 confirmed the activity of DBHB on QS and not on the bioluminescence production. Indeed, the strain *V. harveyi* JAF548 produces bioluminescence which is not under the control of QS. The absence of inhibition of bioluminescence validated the interaction of DBHB with QS [60].

Five steps of QS inhibitions were described, (i) compounds can interact on the biosynthesis of AHL; (ii) compounds can degrade QS signal molecules; (iii) compounds can inhibit AHL efflux protein; (iv) compounds can interact on transcriptional activator or (v) compounds can be considered as QS analogues [61]. It is possible that DBHB can interact on one of four of the last steps described.

DBHB is not the first derivative of compounds from sponges which presents an activity on QS. Another group of marine-sponge-derived compounds was demonstrated to be quorum sensing inhibitors: the manoalide family (Table 2). Extracts from the marine sponge *Luffariella variabilis* were shown to act as quorum sensing inhibitors [62], but the site of action has never been described before for these molecules. It is difficult to define precisely the action of a molecule on the QS because a lot of interactions are possible in the cell. Until now, some natural compounds were described as quorum sensing inhibitors; most compounds identified were able to inhibit the QS receptor such as manoalide or sceptrin type compounds [59]. To find new anti-quorum sensing compounds is an interesting way to disturb microbial communication linked in microfouling [63]. Some studies explained that they are links between QS and biofilm formation, but other studies described that too many pathways are involved to show a direct effect of QS on biofilm [64]. With genomic tools, it will be possible to determine the way of action of the molecule.

DBHB has already been described as an antifouling compound; the molecule can inhibit macrofoulers colonization. Its activity on an enzyme, the phenoloxydase, which occurs in the mussel adhesion, was described (IC_50_ = 0.8 µM) [28]. The compound was also identified as an inhibitor of the barnacle adhesion (10 µM) [29]. In this study, DBHB is able to inhibit bacterial biofilm maturation (PAO1: IC_50_ = 10.2 µM and 4M6: IC_50_ < 2 µM) without toxicity, a possible explanation is that DBHB can interact on bacterial communication. Comparatively to macrofoulers, Gram-negative bacteria were less sensitive than mussels at DBHB, the IC_50_ to inhibit biofilm maturation is 2 at 10 times higher than the enzymatic activity. All these data make DBHB a promising antifouling agent.

## 4. Materials and Methods

All chemical products were purchased from Sigma Aldrich except the syto9^®^ which was purchased in Fisher scientific. DBHB had been synthesized at the Institute of pharmaceutical biology and biotechnology (Heinrich-Heine-University Düsseldorf, Germany) [27].

### 4.1. Bacterial Strains and Growth Conditions

Marine bacteria selected for this study were *Paracoccus* sp. 4M6 (Gram negative, 1 OD = 4.8 × 10^7^ CFU/mL), *Pseudoalteromonas* sp. 5M6 (Gram negative, 1 OD = 3.5 × 10^7^ CFU/mL) and *Bacillus* sp. 4J6 (Gram positive, 1 OD = 4.2 × 10^7^ CFU/mL). These marine bacteria were isolated from glass slides immersed for 6 h in the Morbihan gulf, France [65]. *Paracoccus* sp. 4M6 was selected for its ability to produce bacterial signal molecules: acyl homoserine lactone (AHL). In contrast, *Pseudoalteromonas* sp. 5M6 was chosen as a model species as it does not produce any AHL. Finally, *Bacillus* sp. 4J6 was selected as a representative of Gram positive bacteria. The three strains were maintained and cultivated at 20 °C using Zobell media (ASW 30 g/L, Tryptone 4 g/L, Yeast extract 1 g/L). Bacterial cultures were incubated at 10^6^ UFC/mL for 48 h under agitation.

The biosensor *Vibrio harveyi* JAF548 was used to validate the absence of activity of DBHB on bioluminescence production [66]. The strain was grown in MB medium (37.5 g/L) supplemented with kanamycin at 30 µg/mL.

Two terrestrial bacterial strains were also selected. *Pseudomonas aeruginosa* PAO1 to evaluate antibiofilm activity (1 OD = 2.7.10^8^ CFU/mL), because it is a bacterium well known for biofilm formation and communication processes. The second terrestrial bacterium is the biosensor *Escherichia coli* pSB401 used to describe the anti-quorum sensing property. Terrestrial strains were maintained and cultivated at 37 °C in Luria Bertani, LB (NaCl 10 g/L, Tryptone 10 g/L, Yeast extract 5 g/L) for 24 h under agitation.

All experiments (anti-adhesion, anti-biofilm activities) were realized using *Paracoccus* sp. 4M6 and *P. aeruginosa* PAO1, however the remaining marine bacteria (*Pseudoalteromonas* sp. 5M6, *Bacillus* sp. 4J6, *V. harveyi* JAF548 and *E. coli* pSB401) were used to complete the activity of the DBHB on different bacteria.

### 4.2. Impact of DBHB on AHL Production

AHL was extracted and identified in four strains: *Pseudomonas aeruginosa* PAO1, *Paracoccus* sp. 4M6, *Bacillus* sp. 4J6 and *Pseudoalteromonas* sp. 5M6.

AHL extraction and identification processes were performed using the methodology described by Morin et al. [35]. The effect of DBHB was evaluated at three concentrations (16, 32 and 80 µM). In cultures with and without DBHB, AHL were extracted with dichloromethane in bacterial supernatants. The organic phase was dried and AHL were suspended with acetonitrile. Extracts were injected in LC–MS (microTOF-QII, Bruker, Billerica, MA, USA) and AHL were identified by analysis of the retention time and spectrum obtained [67]. All extractions were realized in triplicate.

### 4.3. Anti-Bacterial Activity

DBHB anti-bacterial activity was evaluated at six concentrations: 0.02, 0.08, 0.2, 2, 16 and 80 µM. Bioassays were run in twelve replicates. The screening was performed on *Paracoccus* sp. 4M6, *Pseudoalteromonas* sp. 5M6 and *Pseudomonas aeruginosa* PAO1. Bacterial cultures of 24 h for PAO1 and 48 h for 4M6 and 5M6 were diluted at 10^6^ CFU/mL and added the microplate containing DBHB to a final volume of 200 µL in a 96-welle microplate in polypropylene. DBHB was added in the first step to allow the evaporation of the solvent (methanol). A positive control consisted of bacteria without DBHB and for the negative control DCOIT (4,5-Dichloro-2-octyl-4-isothiazolin-3-one) was added at 0.1 µg/mL. Microplates were incubated at 20 °C for 48 h for 4M6 and 5M6, and at 37 °C for 24 h for PAO1.

After the incubation the optical density at 600 nm was measured to determine the impact of DBHB on bacterial growth. By a comparison to the control, a bacterial inhibition percentage was determined.

### 4.4. Anti-Adhesion Activities

**Figure 8:**
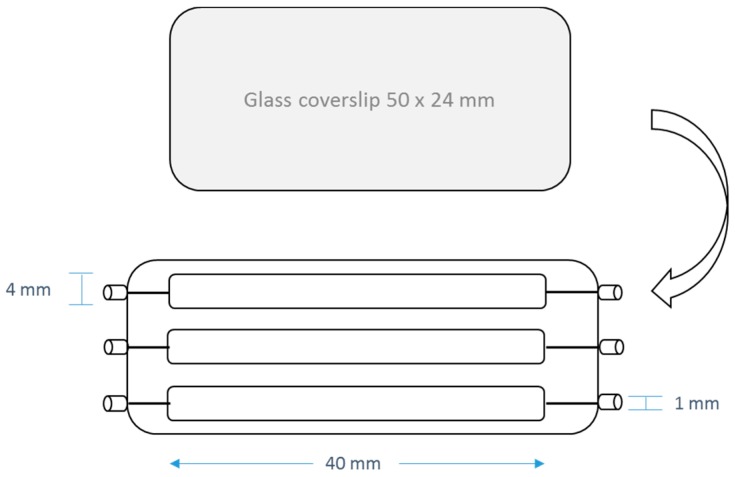
Schematization of the flowcell.

In order to evaluate the anti-adhesion properties of DBHB, experiments were realized in a flowcell [30]. The flowcell was prepared by sticking a microscope coverslip 24 × 50 mm^2^ (VWR) slide which is the support of adhesion. The flowcell is presented on the Figure 8.

The system was sterilized by a flow of bleach (1.5%) for 24 h at 430 µL/min. Then a flow of minimum medium, a saline solution (NaCl 9 g/L) for the terrestrial bacterium: *Pseudomonas aeruginosa* PAO1 or an artificial sea water (ASW 30 g/L) for marine bacteria (*Paracoccus* sp. 4M6, *Pseudoalteromonas* sp. 5M6 and *Bacillus* sp. 4J6) was activated to clean and prepare the system for the bacterial injection at 150 µL/min.

The bacterial solution was prepared from a bacterial culture which was inoculated overnight. A dilution of the bacterial suspension was realized to inject bacteria at 10^6^ CFU/mL in minimum medium. Using a 1 mL syringe, 250 µL of the inoculum was injected in every channel. The flowcell was turned over to facilitate the bacterial adhesion on the microscope coverslip. Bacteria were allowed to attach to the glass surface during 2 h in static condition. For PAO1, the flowcell was incubated at 37 °C, and for 4M6 the flowcell was incubated at 20 °C.

To quantify the anti-adhesion activity, two assays were performed. Firstly, DBHB was added in the flowcell at 16 µM in the bacterial inoculum immediately prior to injection (Condition 1 in Table 5). The concentration of DBHB was determined from results obtained by a screening against bacteria and microalgae (results are not presented). We decided to evaluate the activity of DBHB in lower concentration at 16 µM due to a limitation of DBHB quantity available at the time. Also, to evaluate DBHB activity on bacterial adhesion, the molecule was used to prepare the support of adhesion. DBHB was added at 16 µM in the minimal medium flow. It was used to clean the surface during two h at 150 µL/min (Condition 2 in Table 5). A condition without addition of DBHB was used as a control.

After two hours of adhesion, the flow at 150 µL/min was activated during 30 min with the aim to remove free bacteria. Adhered bacteria were observed with Syto9^®^ nucleic acid stain at 5 µM (λ_excitation_ = 488 nm, λ_emission_ = 498–540 nm). The bacterial adhesion was observed with confocal laser scanning microscopy (Zeiss, LSM 710, Oberkochen, Germany) by using a 40× oil immersion objective. For every studied parameter, a triplicate was realized (27 observations per condition). The overlap percentage was determined with a JAVA program established in the lab. This program determines a percentage between black and green pixels.

### 4.5. Anti-Biofilm Acitivities

In order to evaluate the anti-biofilm property of DBHB on three marine bacteria (*Paracoccus* sp. 4M6, *Pseudoalteromonas* sp. 5M6 and *Bacillus* sp. 4J6) and on one terrestrial bacterium *P. aeruginosa* PAO1, a test in a flowcell was performed. To observe the biofilm formation, a flow of medium growth at 120 µL/min (LB for *P. aeruginosa* PAO1 or Zobell for *Paracoccus* sp. 4M6, *Pseudoalteromonas* sp. 5M6 and *Bacillus* sp. 4J6) was activated during 24 h for *P. aeruginosa* PAO1 or 48 h for marine bacteria to allow the biofilm formation under dynamic condition. Then, bacteria were stained with the same procedure as the adhesion part with syto9^®^ and the biofilm was observed by confocal laser scanning microscopy. The biovolume and the thickness of biofilms were determined with the COMSTAT program [68].

To highlight a potential anti-biofilm property of DBHB, two tests were realized. Firstly, DBHB activity on the biofilm formation was assessed. In this case, DBHB was added to the growth medium flow at 2, 8, 16 and 32 µM. Secondly, DBHB activity on the biofilm degradation was examined. For this second test, DBHB in solution at 16 µM in growth medium was injected in a channel with a formed biofilm. DBHB was left in contact with the biofilm for 2 h and the flow was activated to clean the channel before CLSM observations. The inhibition factor was calculated by a ratio between the percentage of biofilm between the control (bacteria without DBHB, 100%) and the percentage of biofilm obtained by the addition of the compound. For every concentration, a triplicate is realized (27 observations per condition).

The bacterial viability was measured using a double tagging by syto9^®^ nucleic acid stain and sytox red^®^ nucleic acid stain (λ_excitation_ = 633 nm, λ_emission_ = 645–695 nm). The viability percentage was determined from biofilm biovolume.

### 4.6. Anti-Quorum Sensing Activities

In order to measure and characterize DBHB potential effect on QS, a biosensor was used. This biosensor constituted of a strain of *Escherichia coli* containing the plasmid pSB401. This plasmid harbors a *lux*CDABE gene which induces a luminescence when the promotor is activated in presence of AHL, the C_6_-HSL at 500 ng/mL. The selection of *E. coli* containing the plasmid pSB401 was controlled by a resistant gene at the tetracycline (10 µg/mL).

The test was carried out using a polypropylene 96-well black microplate. C_6_-HSL was used at 500 ng/mL to activate the biosensor; acetonitrile was used as carrier solvent for the AHL and was eliminated by evaporation under a laminar flow hood for 30 min. The 96-well was inoculated with *E. coli* pSB401 at 10^6^ UFC/mL in LB media with tetracycline at 10 µg/mL. DBHB was added at 8 and 16 µM to obtain a final volume of 200 µL per well. The 96-well microplate was incubated at 30 °C under agitation for 8 h. Every hour the Optical Density at 600 nm and the luminescence were measured (TECAN, infinite M200pro). A known inhibitor of quorum sensing, kojic acid, was also tested at 35 µM [59].

In order to validate the absence of bioluminescence production inhibition, another biosensor was used: *Vibrio harveyi* JAF548 was mutated on *luxO*D47E, the protein LuxO was maintained in a phosphorylated stage which allows continuous bioluminescence production. In this biosensor, the bioluminescence production is not controlled by quorum sensing and it is activated in continuous.

To evaluate the activity of DBHB, the bioluminescence emission reported to the optical density was measured every hour for twelve hours. A total of 100 µL of AB medium [69] containing the kanamycin at 30 µg/mL, *Vibrio harveyi* JAF548 at 10^6^ UFC/mL and DBHB evaluated at 2 and 8 µM were dropped. The experiment was performed in polypropylene 96-well black microplate.

All experiments were run in six replicates.

### 4.7. Statistical Analysis of Data

The means are reported ± standard deviation (SD). The effect of the compound DBHB on bacterial adhesion, biofilm formation, biofilm degradation, bacterial viability and bacterial communication were tested using the one-factor analysis variance (ANOVA) and the Tukey test (HSD). The level of significance was set to *p* < 0.01. Values are means ± standard deviation.

## 5. Conclusions

It was already shown that bacterial signal molecules such as AHL are involved in relationships between bacteria and other organisms, inlcuding microalgae [70] and macroorganisms (*Ulva* spores, *Balanus* cyprids) [71]. Targeting the bacterial communication system is an interesting strategy to inhibit, first, the microfouling by disruption of bacteria and diatom biofilms and, second, the macrofouling by the reduction of macrofoulers attachment. Dibromohemibastadin is a promising antifouling compound and we can now describe a first approach of this compound for the inhibition of microfouling.

In order to understand the way of action for this compound, a study on biofilm formation and quorum sensing was realized. DBHB was able to inhibit biofilm formation without acting on the adhesion step. The biosensor used, a modified *E. coli* strain, highlighted an interaction of DBHB with bacterial communication. The use of natural or analogue compounds, which have an impact on quorum sensing and biofilm formation, could be an interesting strategy to inhibit microfouling and macrofouling. In addition, the absence of toxicity of DBHB on bacteria is an important factor to consider that would allow DBHB to be applied as an AF agent. To evaluate the activity of DBHB on biofouling, it will be interesting to integrate the compound in a coating and immerse it un-natural environment.

## Figures and Tables

**Table 1:** Identification of acyl-homoserine lactones (AHLs) produced by *P. aeruginosa* PAO1, *Paracoccus* sp. 4M6, *Bacillus* sp. 4J6 and *Pseudoalteromonas* sp. 5M6.

Bacterial Strains	Gram	AHL Produced
*Pseudomonas aeruginosa* PAO1	Negative	C_4_-HSL, C_6_-HSL and 3-oxo-C_12_-HSL
*Paracoccus* sp. 4M6	Negative	C_4_-HSL, C_6_-HSL, C_8_-HSL and 3-oxo-C_10_-HSL
*Bacillus* sp. 4J6	Positive	Autoinducer-2
*Pseudoalteromonas* sp. 5M6	Negative	No. AHL

**Table 2:** Biovolumes of Bacillus sp. 4J6 and *Pseudoalteromonas* sp. 5M6 biofilms calculated for the control condition and after addition of DBHB at 16 and 32 µM in the growth medium flow; the ± represents the standard deviation.

Bacterial strains	Control	16 µM DBHB	32 µM DBHB
4J6 biovolumes (µm^3^/µm^2^)	18.9 ± 1.8	21.5 ± 1.9	22.5 ± 1.7
5M6 biovolumes (µm^3^/µm^2^)	22.0 ± 1.5	21.9 ± 1.3	22.1 ± 1.8
ANOVA		-	-
TUKEY		-	-

-: no statistical difference, *p* > 0.05.

**Table 3:** Biovolumes of PAO1 and 4M6 biofilms tagged with syto9^®^ and sytoxRed^®^ to observe the impact of DBHB at 32 µM on the bacterial viability, the ± represents the standard deviation.

Biovolumes (µm^3^/µm^2^)	*Paracoccus* sp. 4M6		*Pseudomonas aeruginosa* PAO1	
	Control	32 µM DBHB	Control	32 µM DBHB
Living cells (Syto9^®^)	12.7 ± 1.3	4.9 ± 0.1	14.7 ± 1.0	4.3 ± 1.0
Dead cells (SytoxRed^®^)	0.2 ± 0.1	0.3 ± 0.1	0.2 ± 0.02	0.2 ± 0.03
Mortality percentage	0.98	0.94	0.98	0.95
ANOVA		-		-
TUKEY		-		-

-: no statistical difference, *p* > 0.05.

**Table 4:** Antibiofilm compounds derived from marine sponges without killing bacteria or disrupting their growth.

Classes	Compounds	Activity	References
**Terpenoids**	Ageloxime-D	antibiofilm, antimacrofouling	[17,46]
	Manoalide	antibiotic, anti-inflammatory, anti-quorum sensing	[48]
**Pyrrole-imidazole alkaloids**	Oroidin	antibiofilm, antifouling	[49,50,51]
	Sceptrin	antifouling, anti-quorum sensing	[52]
	Bromoageliferin	anti-adhesion, antibiofilm	[47,53]

**Table 5:** Anti-adhesion and anti-biofilm conditions evaluated with DBHB.

Activities	Concentrations	Bacteria	Conditions	
Anti-adhesion	16 µM	*P. aeruginosa* PAO1 *Paracoccus* sp. 4M6	1	Addition in the bacterial suspension
			2	Conditioning of the adhesion surface
Anti-biofilm	2, 8, 16 and 32 µM	*P. aeruginosa* PAO1 *Paracoccus* sp. 4M6	Biofilm formation: addition in the growth medium flow (dynamic: 150 µL/min)	
	16 and 32 µM	*Pseudoalteromonas* sp. 5M6 *Bacillus* sp. 4J6		
	16 µM	*P. aeruginosa* PAO1 *Paracoccus* sp. 4M6	Biofilm degradation: injection of media containing the compound on a biofilm formed (static: 2 h)	
